# Identifying Mental Health Risk in Medical Students: A Quantitative Analysis of Wellness Assessments for First- and Third-Year Students at the University of Ottawa

**DOI:** 10.12688/mep.21343.1

**Published:** 2025-11-12

**Authors:** Kay-Ann Haykal, Inès Zombre, Selena Laprade, Miryam Duquet, Joseph Abdulnour

**Affiliations:** 1 Department of Family Medicine, University of Ottawa, Ottawa, Ontario, K1G 5Z3, Canada; 2 Institut du Savoir Montfort, Hôpital Montfort, Ottawa, Ontario, K1K 0T2, Canada; 3 Faculty of Medicine, University of Ottawa, Ottawa, Ontario, Canada

**Keywords:** Wellness Assessment; Medical Students; Mental Health; Risk Factors; Longitudinal Study

## Abstract

**Introduction:**

An alarming prevalence of burnout among medical students has been reported in many countries, including Canada. To design resilience and wellness programs, it is important to explore individual risk factors. This article presents an example of a Wellness Assessment Program for medical students at the University of Ottawa. The goal was to identify risk factors for poorer mental health outcomes among medical students at the University of Ottawa.

**Methods:**

We conducted a quantitative study using a Wellness Assessment Questionnaire among four cohorts of first- and third-year students.

**Results:**

Risk factors that significantly impacted the mental health of 1 ^st ^and 3 ^rd^ year medical students at the University of Ottawa were physical health, sleep/fatigue, social support, education and career, stress, and drug and/or alcohol use. Students who were originally from Ottawa had more social support and less stress and drug and/or alcohol use (p < 0.001;p=0.009). Being in the Francophone cohort had a positive effect on physical health, but a negative effect on psychological/emotional health (p=0.039;p=0.004). There was a statistically significant difference (p=0.021) between the psychological/emotional health of 1st-year students (M=0.7895) and 3rd-year students (M=0.8923) when covariates (risk factors) were not considered.

**Discussion:**

In the current context of the limited effectiveness of measures to address the negative impacts of medical education on student well-being, this study showed that efficient use of the Wellness Assessment Program data can identify risk factors that have a significant impact on wellness.

## Introduction

It is well known that medical schools are considered stressful environments, as the academic expectations are extreme ^1^. However, it is only recently that the epidemic of burnout related to medical training has been studied ^1^. Medical students face multiple stressors, including high academic demands, emotional tolls from patient care, and the pressure of future career uncertainty, all of which place them at high risk for burnout and poor mental health ^2, 3^. Several studies have shown the negative impacts of medical education on student well-being in several countries, including Canada ^1, 4, 5^. Medical students experience heightened emotional exhaustion and dissatisfaction with work-life balance compared to other graduate students or professionals ^6^. It is therefore not surprising that the prevalence of psychological distress among medical students is significantly higher than in the general population ^7, 8^. Furthermore, when compared to individuals with professional degrees or Doctoral degrees in other fields, physicians experience higher rates of emotional exhaustion and burnout and report more dissatisfaction with work-life balance ^9^. The more years a physician has been in practice, the more positive the perception of well-being, whereas physicians with fewer years (< 5 years) of practice reported greater burnout and lower resilience ^10^. Females had higher odds of having low resilience, and they reported more burnout, depression and suicidal ideation when compared to men ^10^. 

The alarming condition of medical students’ well-being has prompted several institutional organizations, including medical schools, to develop and implement various wellness programs aimed at preventing burnout and promoting resiliency ^11, 12^. However, the targeted risk factors and overall quality of these programs are inconsistent, and there is limited evidence about their effectiveness ^12^. Despite the increasing number of studies on the worrying state of medical students' well-being, few systematic studies have addressed different risk factors reported to date ^12^. Understanding the factors that contribute to medical student distress and how to address them requires identifying risk factors for poor mental health ^12^. The overall goal of this study is to identify risk factors for mental health among undergraduate medical students at the University of Ottawa. Specific objectives are:

1) Identify and assess key risk factors that contribute to mental health distress among undergraduate medical students at the University of Ottawa; 2) Examine how mental health varies between students in the 1st and 3rd year of their undergraduate medical program, including any shifts in risk factors during this time; and 3) Analyze the impact of demographic variables (e.g., gender, age, previous education) on mental health outcomes, with a focus on identifying high-risk groups.

## Methods

### Study design and setting

Students (n=911) from 4 different cohorts completed the Wellness Assessment Questionnaire. As 6 students did not consent to the collection of information, the final sample size was 905, and all of these participants provided written informed consent for their participation in the study. For the purpose of anonymizing the participating years, cohorts were identified with numbers: MD-A(1st year only), MD-B(1st year only), MD-C (1st and 3rd year) and MD-D (1st and 3rd year). 

The sample for this study consists of medical students from 4 different cohorts at the University of Ottawa. The study participants were MD-A 167 students; MD-B 168 students; MD-C 173 students; and MD-D 161 students. Participation in the study was mandatory for all students in their 1st and 3rd years. Students completed the Wellness Assessment Questionnaire as part of the University's Wellness Assessment Program, which has been in place since the 2010–2011 academic year. The data was collected in the academic years 2017–2018 and beyond.

We used a quantitative approach to analyze the Wellness Assessment Questionnaire results among 1 ^st^ and 3rd-year students. The responses were converted to numerical values and analyzed statistically. The rationale for converting the Likert scale responses to a numerical scale is to enable quantitative analysis and statistical processing of the data. While Likert scale items indicate levels of agreement or frequency (e.g., Strongly Disagree, Disagree, Neutral, Agree, Strongly Agree), they are ordinal, meaning they represent an ordered sequence but do not provide the precise distance between each point. This numerical scale facilitates comparisons across participants and enables the aggregation of data in a way that enhances the interpretation and presentation of results, making it easier to conclude trends in wellness, depression, and other factors being assessed.

### Description of the University of Ottawa’s Wellness Assessment Program

The health and well-being of students are a priority for the Faculty of Medicine at the University of Ottawa. The Wellness Assessment Program aims to promote and ensure the health and well-being of students while fostering academic success. It offers a full range of services to undergraduate students, such as personal wellness, psychological health, financial and career planning, including support for the residency application process ^13^. The Wellness Assessment Questionnaire is part of the program, and it is based on the Medical Students Well-Being Index, the Perceived Wellness Survey and the Burns Depression Inventory ^12, 14, 15^. Although not validated, this instrument, collectively, aims to provide a comprehensive assessment of medical students' well-being. The focus is on different dimensions of health—emotional, social, physical, and psychological—allowing the medical program to identify students at risk and offer tailored support through counseling and other resources. The data gathered from these tools is used to improve the wellness initiatives at the University of Ottawa, with the goal of fostering academic success while prioritizing student health. The questionnaire has the following headings: demographic information, mental health and risk factors such as physical health, sleep, social support, financial stress, education and career, stress and drug use and/or alcohol. Open-ended questions are also asked about any concerns the student may have, as well as a list of suggestions for the medical program. 

The wellness Assessment Program is a mandatory assessment of the student wellness conducted in year 1 and year 3 of their undergraduate training. The Wellness Assessment Program was established in 2010–2011, but has been validated. Students complete an online 15-minute Wellness Assessment Questionnaire on Survey Monkey before their appointment, followed by an individual virtual 30-minute meeting with a counsellor. During and following these sessions, students are oriented to the different resources provided in relation to the pillars of the Student Affairs Office (SAO) services. As of the 2017–2018 academic year, the Wellness Assessment Program became a mandatory program for 1 ^st^ year students, and since September 2017, the Wellness Assessment Program has been mandatory and since then, all students must complete the questionnaire.

Students in year 3 of clerkship are also invited to participate in the Wellness Assessment Program voluntarily. An email is sent to all students at the end of April of each year to complete the survey online, accompanied by an invitation to follow up with an in-person visit with a counsellor to address any concerns or to build strengths. Counsellors communicate with students if they identify specific concerns or red flags upon reviews of the surveys completed as well. As of the academic year 2017–2018, the 3 ^rd^ year Wellness Assessment Program became mandatory as well ^16^.

### Data analysis

We analyzed the responses to the Wellness Assessment Questionnaire of the four cohorts MD-A (1st year only), MD-B (1st year only), MD-C (1st and 3rd year) and MD-D (1st and 3rd year), with Likert scales and converted to numbers to perform a statistical analysis addressing different questions. The data were longitudinal for cohorts MD-C and MD-D. A multiple linear regression analysis was performed to identify independent predictors of psychological/emotional health. An Independent sample t-test was used to compare various risk factors of mental health between gender, study cohort (francophone vs. anglophone) and residence. Linear mixed model repeated measures were performed to determine if time (1st year to 3rd year) affects the psychological/emotional health of the students. A second linear mixed model with covariates was also performed to determine if time still affects psychological/emotional health. 

Data were converted from the Likert scale to a numerical scale (Strongly Disagree = -2, Disagree = -1, Neutral = 0, Agree = 1, Strongly Agree = 2).

The data for some questions were also reversed so that a high score in each category was considered "healthy" and a low score was considered "unhealthy". For example, for question 13: *In general, I have good eating habits*, a Strongly Disagree has a low score (-2) while a Strongly Agree response has a high score (2). Therefore, the healthier the student is physically, the higher the score and vice versa.

On the other hand, question 38: *I rarely engage in exercise and/or physical activities* was reversed. Therefore, a student who answered Strongly Disagree had a score of 2 (they are in better physical health), and a student who answered Strongly Agree had a score of -2 (they are in poor physical health). Due to the different number of questions by categories in the 1 ^st^- and 3 ^rd^-year questionnaire, we had to use the average of the responses to the questions in each category for each student.

SPSS 22.0 for Windows was used to perform statistical analyses. Results are expressed as means ± standard deviation and standardized beta coefficient with a 95% confidence interval. A p-value ≤ 0.05 was considered statistically significant.

## Results

The study identified several risk factors for mental health among medical students, differing across the first and third years. For first-year students, significant predictors included sleep/fatigue, social support, education and career pressures, and stress combined with substance use, all with positive associations to mental health scores (p < 0.001). In the third year, these factors remained significant, with the addition of physical health as a predictor. Interestingly, financial challenges negatively impacted mental health in the third year (p=0.011). When combining responses from both years, sleep/fatigue, social support, education/career, and stress/substance use consistently influenced mental health outcomes. Overall, the predictive model explained between 56% and 73% of the variance in psychological/emotional health scores across these groups.

The results also revealed variations in mental health based on gender, cohort, and residence. Males reported better psychological/emotional health, sleep, and stress management compared to females (p < 0.05), while females experienced more significant challenges in these areas. Francophone students had slightly better physical health but poorer psychological/emotional health than their Anglophone peers (p=0.004). Students from Ottawa reported higher levels of social support compared to those from out-of-town (p < 0.001), though there were no significant differences in their overall mental health scores. Furthermore, third-year students showed marginally better mental health outcomes than first-year students (p=0.021), suggesting potential adaptation or resilience over time despite persistent stressors.

### Risk factors for medical health among medical students

Analyses were performed on three groups: 1 ^st^-year students only, 3rd-year students only, and all responses combined (1st-year responses from MD-A, MD-B, MD-C, and MD-D, and 3rd-year responses from MD-C and MD-D). At the time of conducting this study, only two cohorts had completed both year 1 and year 3 assessments.

A multiple linear regression was calculated to determine predictors of the psychological/emotional health score. The model was a significant predictor explaining 56% (R square = 0.556; p < 0.001), 73% (R square = 0.726; p < 0.001) and 59% (R square = 0.594; p < 0.001) for the 1 ^st^ year, 3 ^rd^ year and both years, respectively. Significant predictors are presented in Table 1.

**Table 1.** Mental health risk factors for 1 ^st^, 3 ^rd^ and all responses (1 ^st^ and 3 ^rd^ year).

	B	Standard Error	95% Confidence Interval		Standardized coefficient Beta	p value
			Lower Limit	Upper limit		
**Year 1**						
Sleep/fatigue	0.18	0.03	0.125	0.237	0.24	< 0.001
Social support	0.30	0.04	0.226	0.375	0.28	< 0.001
Education and Career	0.19	0.05	0.098	0.285	0.14	< 0.001
Stress and drug and/or alcohol use	0.31	0.05	0.219	0.394	0.24	< 0.001
**Year 3**						
Physical health	0.13	0.04	0.056	0.197	0.13	< 0.001
Sleep/fatigue	0.20	0.03	0.138	0.267	0.27	< 0.001
Finance	-0.06	0.02	-0.108	-0.014	-0.09	0.011
Education and Career	0.33	0.05	0.233	0.431	0.30	< 0.001
Stress and drug and/or alcohol use	0.36	0.05	0.252	0.465	0.30	< 0.001
**Year 1 & 3**						
Physical health	0.05	0.03	0.005	0.102	0.06	0.030
Sleep/fatigue	0.21	0.02	0.162	0.249	0.27	< 0.001
Social support	0.17	0.03	0.116	0.228	0.17	< 0.001
Education and Career	0.30	0.03	0.229	0.363	0.24	< 0.001
Stress and drug and/or alcohol use	0.30	0.04	0.226	0.363	0.24	< 0.001

B = Unstandardized Coefficient. Regression method. Variables entered in the model for each group year: Are you new to Ottawa; MD program stream (French or English); Gender; Physical health; Sleep/fatigue; Social support; Finance; Education and career; Stress and drug and/or alcohol use. ***Relation between risk factors and mental health based on gender, cohort (Francophone vs. Anglophone) and whether the student is coming from Ottawa or out-of-town***

### Mental health in the 1 ^st^ year compared to the 3 ^rd^ year of medical school

The linear mixed model repeated measures analysis conducted showed a statistically significant difference (p=0.021) between 1st-year students (0.79 ± 0.63) and 3 ^rd^ students (0.89 ± 0.59) on psychological/emotional health.

## Interpretation

### Summary of the main results of the study

The results from Table 1 show that the higher the standardized coefficient, the greater impact it has on mental health. For every 1 unit increase of the risk factor, there is an impact (increase in this case) on the mental health value corresponding to the standardized coefficient. Table 2 shows a significant difference between genders in physical health, sleep/fatigue, finance, education and career, stress and drug and/or alcohol use and psychological/emotional health. Compared to males, females have a poorer outcome in terms of physical health, sleep/fatigue, finances, education and career stress and drug/alcohol use, and psychological/emotional health.

**Table 2.** Difference between gender on well-being predictors.

	Average for female (N=519)	Average for male (N=386)	p value
Physical health	0.87 ± 0.65	0.99 ± 0.63	0.007
Sleep/fatigue	0.11 ± 0.83	0.38 ± 0.81	< 0.001
Social support	0.87 ± 0.61	0.93 ± 0.62	0.133
Finance	0.47 ± 0.86	0.62 ± 0.88	0.009
Education and Carreer	0.52 ± 0.48	0.66 ± 0.55	< 0.001
Stress and drug and/or alcohol use	0.84 ± 0.51	0.99 ± 0.52	< 0.001
Psychological/emotional health	0.72 ± 0.62	0.92 ± 0.63	< 0.001

N =905 Data are presented as mean ± standard deviation.

Table 3 shows a significant difference between the language cohort on physical health and psychological/emotional health. Results showed that being in one cohort or the other partially explains the higher (and therefore healthier) scores in these categories. Therefore, being in the Francophone cohort showed a higher positive effect on physical health and a lower psychological/emotional health compared to Anglophones.

**Table 3.** Difference between cohorts on well-being predictors.

	Average for the Francophone cohort (N=296)	Average for the Anglophone cohort (N=609)	p value
Physical health	0.99 ± 0.63	0.89 ± 0.65	0.039
Sleep/fatigue	0.17 ± 0.85	0.25 ± 0.82	0.186
Social support	0.91 ± 0.62	0.89 ± 0.61	0.570
Finance	0.47 ± 0.90	0.56 ± 0.85	0.160
Education and Career	0.55 ± 0.50	0.59 ± 0.52	0.297
Stress and drug and/or alcohol use	0.88 ± 0.50	0.91 ± 0.52	0.428
Psychological/emotional health	0.72 ± 0.65	0.85 ± 0.62	0.004

N = 905 Data are presented as mean ± standard deviation.

Table 4 shows a significant difference between students’ origin on social support and stress and drug and/or alcohol. Coming from Ottawa partially explains the higher (and therefore healthier) scores for these risk factors. This means that students who come from Ottawa have more social support and less stress and drug and/or alcohol use when compared to students coming from out of town.

**Table 4.** Difference related to students’ residence on well-being predictors.

	Average for students coming from Ottawa (N=486)	Average for students coming out-of-town (N=419)	p value
Physical health	0.91 ± 0.64	0.94 ± 0.65	0.552
Sleep/fatigue	0.23 ± 0.83	0.23 ± 0.83	0.992
Social support	0.97 ± 0.60	0.81 ± 0.62	< 0.001
Finance	0.57 ± 0.85	0.48 ± 0.89	0.135
Education and Career	0.58 ± 0.50	0.58 ± 0.52	0.885
Stress and drug and/or alcohol use	0.95 ± 0.53	0.86 ± 0.5	0.009
Psychological/emotional health	0.81 ± 0.64	0.79 ± 0.63	0.638

N = 905 Data are presented as mean ± standard deviation.

Finally, the mental health of the 1 ^st^ year students is poorer when compared to the 3 ^rd^ year students. However, when considering the covariates (physical health, sleep/fatigue, social support, finances, education and career, and stress and substance use), the difference is no longer significant.

### Findings explanation

Our study used a Wellness Assessment Questionnaire, which is part of the University of Ottawa Wellness Assessment Program, to determine risk factors for mental health among medical students. It is acknowledged that poor mental health of medical students is a “disease condition that is endemic to medical schools” ^17^. A systematic overview of risk factors for suicidal ideation and suicide attempts among medical students indicated that poor mental health outcomes, such as burnout and depression, are the strongest risks. Female gender was significantly associated with an increased risk of suicidal ideation, compared with males ^17^. Additionally, strong evidence on the wellness of medical students shows that their well-being is deteriorating throughout training, and it is becoming a growing problem ^18, 19^. In 2016, Canadian medical students reported burnout and suicidal ideation rates of 37% and 6%, respectively ^20^. An increase in the prevalence of mood disorders, anxiety disorders, suicidal ideation, and psychological distress in Canadian medical students compared to age-matched controls from the general population was observed in 2019 ^20^. It is well documented that physicians who demonstrate an elevated level of resilience report better well-being that promotes fulfillment in their work environment ^21^. It is then favorable to support the development of resilience wellness programs in medical training, to equip and prepare students for the challenges of the clinical environment ^18, 22^. However, the lack of enthusiasm for resilience programs in medical education remains. The culture that "doctors are invincible" persists (p.6–7) ^20^. A first systematic review conducted on the effectiveness of resilience programs in medical education indicates an improvement in resilience and well-being across several studies; however, no standardized resilience program has been evaluated ^23^.

The study found significant gender, cohort, and residential differences in medical students' well-being and related factors, with females reporting lower psychological/emotional health, higher stress, and poorer sleep compared to males. Additionally, Francophone students had better physical health but lower psychological health compared to Anglophone students, and students from Ottawa reported higher social support and better stress management than those from out-of-town.

### Future directions

In the current context of limited effectiveness of resources (wellness programs, access to tertiary care services) implemented to address the negative impacts of medical education on student well-being ^17^, this study shows that thoughtful use of the Wellness Assessment Program can identify risk factors that have a significant impact on medical students’ mental health. Wellness programs could also tailor effective interventions to address these risk factors for poor mental health ^24^. In addition to the thoughtful use of Wellness Assessment Program, it is also required to have a comprehensive approach to address the systemic and structural factors in which students live and learn ^25^. Most wellness and resilience programs implemented to date tend to focus on teaching self-care to students, when the main problem often comes from the work environment ^26^. Resilience programs implemented at our Faculty of Medicine, such as mindfulness training, peer support and mentorship programs, and wellness workshops help students manage stress and prevent burnout. These programs focus on self-care, time management, and mental health support, aiming to build emotional resilience and promote work-life balance. Additionally, physical wellness initiatives and cognitive behavioral therapy-based programs support students' well-being. Further studies are needed to explore the influence of the work environment on poor mental health among medical students to evaluate the outcomes of wellness and resilience programs targeting risk factors identified by medical students. This study’s data was gathered in pre-covid context; further studies could consider the impact of COVID-19 on the program.

Furthermore, students have expressed strong support for the mandatory assessment, with many agreeing that it is essential for identifying and addressing mental health and well-being challenges early in their education. They believe it provides an important opportunity for establishing an initial connection with the SAO, proactive intervention and support, particularly in such a high-pressure environment. Given this positive reception, the next steps could include formalizing the assessment within the curriculum and ensuring that it is integrated into students’ regular academic requirements. Additionally, regular feedback and evaluation of the program will be crucial for refining its effectiveness and ensuring it continues to meet students' evolving needs.

### Limitations of the study

The lack of homogeneity in responses across student cohorts may have affected the study's results. There was significant variability in responses regarding risk factors on mental health, as the percentage of impact of risk factors on psychological and emotional health was unequally distributed among students. Also, there were more responses from first-year students considered. Furthermore, given that there is no data on the psychometric properties of the instrument currently used, it is difficult to determine whether the differences arise from real differences among students or from artifacts due to the instrument (e.g. problem loyalty).

In addition, this questionnaire is a mixture of statements from several English instruments, which makes it less reliable for administration to French-speaking students; the translated version does not give rise to a back-translation or validation. The lack of data on the psychometric properties of the instrument used in this study raises significant concerns about the reliability and validity of the results. Without assessing the psychometric properties, such as reliability (e.g., internal consistency, test-retest reliability) and validity (e.g., content, construct, and criterion validity), it is difficult to ensure that the instrument is accurately measuring what it intends to. This gap in psychometric evaluation means that the observed differences in mental health risk factors might not be due to genuine differences among the students but could be artifacts caused by the instrument itself, such as biases in translation or response inconsistencies across cohorts.

Therefore, while the study provides useful insights, the absence of validated tools undermines confidence in the results. To have greater confidence in their findings, the researchers would need to validate the instrument through established psychometric methods, including conducting a thorough analysis of its reliability and validity across different student populations, particularly given the mixed-language context. This step is crucial before drawing firm conclusions or applying these results in a broader context, and supports further research in this area. Finally, the study represents one organization only and hence may not be reproducible.

## Conclusion

This study found that several key risk factors significantly impact the mental health of medical students, including sleep/fatigue, social support, stress, and substance use. Specifically, these factors were strong predictors of psychological and emotional health in both first- and third-year students. It also revealed differences between student groups, such as gender and cohort (Francophone vs. Anglophone), on aspects of well-being, with females reporting poorer psychological health than males, and the Francophone cohort showing lower psychological/emotional health compared to the Anglophone cohort. The alarming condition of medical students’ well-being prompted medical schools to develop and implement various wellness programs for burnout prevention and resilience training. However, resilience training and wellness programs are unlikely to make a difference if they are not used efficiently. This study shows that wellness programs may be relevant to identifying risk factors related to poor mental health among medical students and implementing interventions that are more effective.

## Ethical approval

This project received an ethics exemption from the Ottawa Health Science Network Research Ethics Board as it was determined to fall in the category of quality improvement and that it did not constitute human subject research.

## Data Availability

Our data are confidential and consist of medical student wellness assessments. Because of their sensitive nature, they cannot be shared publicly. The data are securely stored on the University of Ottawa’s institutional shared drive, which complies with the university’s privacy and confidentiality standards. In accordance with the MedEdPublish data availability policy, de-identified or aggregate data may be available upon reasonable request, provided that such sharing is consistent with our institutional ethics approval and privacy regulations. Requests can be directed to Dr. Kay-Anne Haykal by email at khaykal@uottawa.ca, and access will only be granted under conditions that preserve student confidentiality.
