# Supplementary material for: Identifying Mental Health Risk in Medical Students: A Quantitative Analysis of Wellness Assessments for First- and Third-Year Students at the University of Ottawa
Source: MedEdPublish (2016). 2025 Nov 12;15:261. [Version 1] doi: 10.12688/mep.21343.1 (PMC13560868; doi:10.12688/mep.21343.1)
Supplement: Peer review history

# Reviewer report: Reviewer response for version 1

**DOI:** 10.21956/mep.22857.r44728

**Published:** 2025-12-18

**Authors:** Alba Madrid Cagigal, Carmen Kealy

**Affiliations:** 1 University of Galway, Galway, County Galway, Ireland

Thank you for the opportunity to review this interesting and relevant manuscript. I have outlined several areas where clarification or further detail may help strengthen the work.

**Introduction**:

1. Outdated references. Some of the cited studies date from 2010–2019. I would consider incorporating more recent references where available to strengthen the contemporary relevance of the introduction.
2. Clarify rationale behind practice-duration burnout differences. The statement “The more years a physician has been in practice, the more positive the perception of well-being, whereas physicians with fewer years (< 5 years) of practice reported greater burnout and lower resilience” [Page 3, #19] would benefit from a brief explanation or supporting reference to clarify why burnout and resilience differ by years in practice. As such, it would be helpful to mention whether the literature offers any preliminary hypotheses explaining this pattern.
3. Clarify who the “females refer to”. The sentence “Females had higher odds of having low resilience, and they reported more burnout, depression and suicidal ideation when compared to men” [Page 3, #23] needs clarification. Does this refer to female physicians or medical students? I would specify and provide a bit more context.
4. Explicitly connect gaps in wellness programs to study aims. Although the introduction notes that existing wellness programs do not fully address risk factors, the link between these gaps and the rationale for this study could be made more explicit. Expanding on how current programs fall short would strengthen the justification for focusing on risk identification.

**Methods**:

1. Clarify MD-A vs MD-B, and MD-C vs MD-D. The distinctions between MD-A and MD-B, as well as MD-C and MD-D, are unclear [Page 4]. Providing a brief explanation of these cohort labels would improve clarity.
2. Mandatory vs voluntary participation is confusing. The manuscript presents conflicting statements about participation. First it states “participation in the study was mandatory for all students in their 1st and 3rd years” [Page 3] but then it is specified that  “as of the 2017–2018 academic year, the Wellness Assessment Program became a mandatory program for 1st year students, and since September 2017, the Wellness Assessment Program has been mandatory and since then, all students must complete the questionnaire” [Page 4, # 10]. Later, it says “Students in year 3 of clerkship are also invited to participate in the Wellness Assessment Program voluntarily” [Page 4, #15]. This is quite confusing. Please clarify when did the programs become mandatory, for which cohorts, whether some groups were voluntary before certain years, and why different requirements existed. Maybe a short timeline or table could be helpful
3. Clarify opt-out despite ethics exception. Although ethics exemption was granted, it would be helpful to explicitly state whether students could opt out of the assessment without consequences.

**Results**

1. Clarify direction of “positive associations”. The sense describing first-year student’s predictors states that the factors had “positive associations to mental health scores” [Page 4, #5 of Results section]. Please clarify that these factors were associated with worse mental health scores.
2. P-value reporting consistency. Some p values are reported as exact, others as <0.001. I would recommend consistency.

**Discussion**

1. Expand on the “lack of enthusiasm for resilience programs”. The statement “However, the lack of enthusiasm for resilience programs in medical education remains” [Page 7, #30] would benefit from a supporting reference and brief explanation.
2. Cultural framing: Considering the study includes Anglophone and Francophone cohorts, it would be helpful to discuss whether cultural or linguistic differences may have influenced responses, particularly in light of score differences between groups.

Have any limitations of the research been acknowledged?

Yes

Is the work clearly and accurately presented and does it cite the current literature?

Partly

If applicable, is the statistical analysis and its interpretation appropriate?

Yes

Are all the source data underlying the results available to ensure full reproducibility?

Yes

Is the study design appropriate and is the work technically sound?

Yes

Are the conclusions drawn adequately supported by the results?

Yes

Are sufficient details of methods and analysis provided to allow replication by others?

Partly

Reviewer Expertise:

Digital mental health and university students.

We confirm that we have read this submission and believe that we have an appropriate level of expertise to confirm that it is of an acceptable scientific standard, however we have significant reservations, as outlined above.

# Reviewer report: Reviewer response for version 1

**DOI:** 10.21956/mep.22857.r44797

**Published:** 2025-12-16

**Authors:** DAVID PEREZ-JORGE

**Affiliations:** 1 University of La Laguna, San Cristóbal de la Laguna, Spain

Dear Authors,

 Thank you for submitting your manuscript on identifying mental health risk factors among medical students using data from an institutional wellness program. We value the relevance of the topic and the practical potential of your findings (sleep/fatigue, stress, social support, substance use), as well as the differences observed between first- and third-year students, by place of origin and language cohort.

 Following the review, my recommendation is a **major revision**. For a favorable reconsideration, please prioritize the following:

1. **Sample and traceability.** Specify the sample size by year/cohort, inclusion criteria, response rate, and provide a flow diagram of the process (identification–inclusion). Include a descriptive table by subgroups.
2. **Instrument and psychometrics.** Describe the questionnaire structure (constructs and items), provide reliability (α/ω) for each subscale, and, if possible, a structural verification (EFA/CFA with fit indices). Include the instrument in the Supplement.
3. **Analytical plan and multiplicity.** Complement p-values with effect sizes and 95% CIs; implement multivariable models (e.g., linear/logistic regression) adjusting for confounders (age, gender, cohort, origin) and apply a multiplicity control (FDR/Bonferroni)—present sensitivity analyses/stratifications where appropriate.
4. **Clarity and magnitude of results.** For statements such as “positive/negative effect” or differences across years/cohorts, report group means/SDs or medians/IQRs and the corresponding difference (e.g., Hedges’ g/OR) with 95% CIs.
5. **Ethics and data.** Add details of ethics approval/exemption and a data policy (an anonymized aggregated dataset plus a data dictionary/instrument in a repository or available upon reasonable request) to strengthen reproducibility.
6. **Presentation.** Improve the readability of figures/tables (fonts, labels) and standardize terminology.

We appreciate the practical value of your approach (using wellness assessments for screening and prevention). With these enhancements—especially the psychometrics, multivariable analysis with CIs, and multiplicity control—the study will gain in validity and traceability, positioning it well for a favorable decision.

 I look forward to your point-by-point response indicating the page/section/table/figure where each change is implemented.

 Sincerely

Have any limitations of the research been acknowledged?

Partly

Is the work clearly and accurately presented and does it cite the current literature?

Partly

If applicable, is the statistical analysis and its interpretation appropriate?

Partly

Are all the source data underlying the results available to ensure full reproducibility?

Partly

Is the study design appropriate and is the work technically sound?

Partly

Are the conclusions drawn adequately supported by the results?

Partly

Are sufficient details of methods and analysis provided to allow replication by others?

Partly

Reviewer Expertise:

Publich health, Education health

I confirm that I have read this submission and believe that I have an appropriate level of expertise to state that I do not consider it to be of an acceptable scientific standard, for reasons outlined above.

# Reviewer report: Reviewer response for version 1

**DOI:** 10.21956/mep.22857.r44631

**Published:** 2025-12-11

**Authors:** Alexandre Hudon

**Affiliations:** 1 Universite de Montreal, Montreal, Québec, Canada

**Brief summary**

 This quantitative study analyzes mandatory wellness assessments of first- and third-year medical students at the University of Ottawa to identify risk factors linked with poorer mental health.

 Please see my comments below.

**Introduction**

- The introduction is thorough but could be tightened by reducing repetition (multiple statements about high rates of burnout and distress). Consider streamlining to improve focus.
- The rationale for focusing specifically on Ottawa’s undergraduate cohorts would benefit from clearer justification, as the introduction currently presents a global problem without fully linking it to the local context.
- Some cited statistics are strong but could be more directly connected to the study objectives, making the narrative more cohesive.
- The objectives are listed clearly but could be framed earlier in the introduction to anchor the reader before the detailed background.
- Consider briefly previewing the role and limitations of the Wellness Assessment Program earlier, since this contextualizes the later methodological constraints.

**Methods**

- Expand on the Wellness Assessment Questionnaire’s lack of validation and discuss how this may influence data interpretation; this is acknowledged later in the limitations but would be helpful upfront.
- Provide more detailed descriptions of each category ( “stress and drug/alcohol use”), such as example items, to improve reproducibility.
- Clarify how mandatory vs. voluntary participation was handled in earlier cohorts and whether this influenced response rates or biases.
- The conversion of Likert scales into numerical scores is described, but more justification for treating the resulting variables as continuous would strengthen the statistical rigor.
- More details on model diagnostics and assumptions for regression and mixed models (normality, multicollinearity, etc.) would enhance transparency.

**Results**

- Strengthen clarity by consistently reporting effect sizes (standardized coefficients) alongside p-values in the narrative, not only in tables.
- Consider reorganizing results into thematic subsections ( “Predictors of mental health”, “Gender differences”, “Language-cohort differences”) to improve readability, as the current flow is dense.
- Include brief interpretations alongside key numeric findings to aid comprehension, especially when describing tables 1-4.
- Ensure consistency in terminology (“psychological/emotional health” vs. “mental health”) to avoid ambiguity.
- Figures or visual summaries could improve accessibility, given the large number of variables and comparisons.

**Discussion**

- The discussion summarizes existing literature well, but the link between the study’s findings and prior evidence could be made more explicit, especially regarding gender and language differences.
- Some claims could benefit from improved precision; for example, it would help to clarify whether poorer outcomes among Francophone students might be related to linguistic, cultural, or programmatic factors.
- The discussion occasionally shifts into broader commentary on wellness programs without directly tying those points back to the study's findings. Stronger integration would improve focus.
- Future directions could more explicitly recommend validating the assessment tool, given the major limitations associated with psychometric uncertainty.
- Consider expanding on how the COVID-19 context (mentioned briefly) might alter results or generalizability, since data were pre-pandemic.

Have any limitations of the research been acknowledged?

Yes

Is the work clearly and accurately presented and does it cite the current literature?

Yes

If applicable, is the statistical analysis and its interpretation appropriate?

Yes

Are all the source data underlying the results available to ensure full reproducibility?

Yes

Is the study design appropriate and is the work technically sound?

Yes

Are the conclusions drawn adequately supported by the results?

Yes

Are sufficient details of methods and analysis provided to allow replication by others?

Yes

Reviewer Expertise:

Medical Education, Mental Health, AI

I confirm that I have read this submission and believe that I have an appropriate level of expertise to confirm that it is of an acceptable scientific standard, however I have significant reservations, as outlined above.
